# Multi-modal texture fusion network for detecting AI-generated images

**DOI:** 10.3389/frai.2025.1663292

**Published:** 2025-10-22

**Authors:** Haozheng Yu, Bing Xu

**Affiliations:** School of Public Policy and Administration, Nanchang University, Nanchang, China

**Keywords:** AI-generated content, image processing, multimedia forensics, texture analysis, multi-modal

## Abstract

With the rapid advancement of AI-generated content, detecting synthetic images has become a critical task in digital forensics and media integrity. In this paper, we propose a novel multi-modal fusion network that leverages complementary texture and content information to improve the detection of AI-generated images. Our approach integrates three input branches: the original RGB image, a local binary pattern (LBP) map to capture micro-texture irregularities, and a gray-level co-occurrence matrix (GLCM) representation to encode statistical texture dependencies. These three streams are processed in parallel through a shared-weight convolutional backbone and subsequently fused at the feature level to enhance discrimination capability. Extensive experiments conducted on benchmark datasets demonstrate that our method outperforms existing single-modality baselines and achieves strong generalization across multiple types of generative models. The proposed fusion framework offers an interpretable and efficient solution for robust and reliable detection of AI-synthesized imagery.

## 1 Introduction

With the rapid development of generative artificial intelligence, particularly deep generative models such as GANs and diffusion models, synthetic images that are highly realistic and visually indistinguishable from authentic ones have become increasingly prevalent (Fan et al., 2024). While these technologies offer significant benefits across various industries (Zhu et al., 2024), they also pose serious security and ethical risks (Lu et al., 2023). AI-generated images can be maliciously exploited to mislead the public, manipulate social media narratives, impersonate individuals, or fabricate evidence in sensitive domains such as journalism, politics, law enforcement, and financial systems. The misuse of such content, especially in the form of deepfakes (Westerlund, 2019; Lin et al., 2024b; Ding et al., 2024b), can erode public trust, incite social unrest, and facilitate criminal activities, including fraud, defamation, and identity theft (Ding et al., 2024a). Therefore, developing reliable and effective methods (Chang et al., 2021) to detect AI-generated images has become an urgent necessity for safeguarding digital media authenticity and ensuring public safety (Duszejko et al., 2025; Epstein et al., 2023).

In response to the growing threat of synthetic media, a wide range of AI-generated image detection methods have been developed in recent years. Early approaches primarily relied on hand-crafted features, such as noise inconsistencies, compression artifacts, or frequency anomalies (Mallet et al., 2025; Liu et al., 2024; Alam et al., 2024). With the rise of deep learning, convolutional neural networks (CNNs) have become the dominant paradigm, enabling automatic feature extraction from spatial and frequency domains. More recent studies have also leveraged vision transformers, multi-modal learning (Ramachandram and Taylor, 2017), and contrastive training (Chuang et al., 2020) to enhance generalization across different generative models. Despite these advancements, most existing detectors still struggle with two key challenges: (1) limited generalizability to unseen generative techniques and data domains, and (2) insufficient sensitivity to subtle texture inconsistencies that often reveal the synthetic nature of AI-generated images. These limitations highlight the need for more robust and interpretable detection frameworks that can effectively exploit both visual content and underlying texture patterns.

Texture analysis has long been a fundamental technique in image processing and digital forensics (Nailon, 2010), offering powerful cues for identifying subtle irregularities that are often imperceptible to the human eye. Two widely used methods for texture representation are the Local Binary Pattern (LBP) (Ojala et al., 2002) and the Gray-Level Co-occurrence Matrix (GLCM) (De Siqueira et al., 2013). LBP encodes local texture by thresholding neighborhood pixels relative to a central pixel, effectively capturing fine-grained micro-patterns that reflect surface roughness and local contrast. GLCM, on the other hand, models the statistical co-occurrence of pixel intensities at specific spatial distances and directions, providing a global measure of textural homogeneity, correlation, and entropy. These descriptors have proven effective in a variety of tasks, including medical image analysis, material classification, and forgery detection. In the context of AI-generated image detection, they offer a complementary perspective to semantic content, enabling models to identify subtle texture inconsistencies introduced during the image synthesis process.

Hence, we propose the multi-modal texture and content fusion network for detecting AI-generated images in this paper. Unlike many existing approaches that rely heavily on large-scale datasets to train end-to-end deep networks, our work emphasizes the importance of leveraging diverse modalities and structural image representations to enhance detection capability. Rather than treating the detection task as a purely data-driven classification problem, we aim to extract and fuse complementary features from multiple perspectives—including semantic content, local texture patterns, and statistical dependencies—to provide a richer and more discriminative feature space. In particular, by integrating Local Binary Pattern (LBP) and Gray-Level Co-occurrence Matrix (GLCM) representations alongside the raw RGB input, our method encourages the network to focus on subtle textural artifacts and latent semantic inconsistencies often introduced during image synthesis. This fusion-based strategy enables more interpretable and robust detection, especially in scenarios where visual content alone may be insufficient to distinguish between real and AI-generated imagery. Our design represents a shift toward texture-aware, multi-modal learning in the field of generative image forensics.

The main contributions of this work are summarized as follows:

- We propose a novel three-branch convolutional network that integrates raw RGB images with Local Binary Pattern (LBP) and Gray-Level Co-occurrence Matrix (GLCM) representations. This design enables the model to jointly learn from semantic content and texture-based features, facilitating more accurate detection of AI-generated images through multi-modal feature fusion.
- We enhance texture-based analysis by tailoring LBP and GLCM representations for AI-generated image detection. These refined descriptors help uncover latent semantic artifacts embedded in the synthesis process, allowing the network to focus on subtle but consistent textural cues indicative of forgery.
- We conduct extensive experiments, including ablation studies, to evaluate the effectiveness of the proposed method and the contribution of each input modality. The results demonstrate the robustness and interpretability of our approach, as well as its superiority over traditional single-modality baselines.

The remainder of this paper is organized as follows. Section 2 briefly reviews related work in AI-generated image detection and texture-based analysis. Section 3 presents our proposed multi-modal detection framework in detail. Section 4 reports and analyzes the experimental results, including ablation studies. Finally, we concludes the paper and discusses potential directions for future research.

## 2 Background

### 2.1 AI-generated image synthesis

Recent advances in generative artificial intelligence have led to the development of powerful models capable of producing highly realistic synthetic images. Notable architectures include Generative Adversarial Networks (GANs) (Ding et al., 2022b), Variational Autoencoders (VAEs) (Kingma et al., 2019), and, more recently, diffusion models (Croitoru et al., 2023). These models can generate high-fidelity human faces (Fan et al., 2025; Ding et al., 2021), objects, or entire scenes that are often indistinguishable from real photographs to the human eye. While such technologies have enabled creative and industrial applications, they also raise serious concerns regarding misinformation, digital impersonation, and the erosion of media trust (Fan et al., 2023).

The task of detecting AI-generated images presents several major challenges (Ye et al., 2024). First, many synthetic images exhibit high visual realism, making it difficult to distinguish them based on low-level visual cues. Second, different generative models leave behind different and often subtle artifacts, requiring detectors to generalize across diverse and evolving synthesis techniques. Third, deepfake detectors may become overfitted to the training distribution and fail on unseen generative methods (Ding et al., 2022a). These challenges demand detection strategies that are robust, generalizable, and capable of capturing subtle and non-obvious visual inconsistencies.

### 2.2 Texture analysis in image processing and digital forensics

Texture is a fundamental visual attribute that captures the spatial arrangement and structural repetition of pixel intensities in an image. Unlike high-level semantic features, which relate to objects or scenes, texture features often encode fine-grained patterns such as surface roughness, regularity, and coarseness (Humeau-Heurtier, 2019). These properties make texture analysis a powerful tool in a wide range of applications, including medical imaging (Lan et al., 2018), material classification, biometric recognition, and image forensics.

Among various texture descriptors, Local Binary Pattern (LBP) and Gray-Level Co-occurrence Matrix (GLCM) have been widely adopted due to their simplicity and effectiveness. LBP encodes the local structure around each pixel by thresholding its neighbors, producing a binary pattern that is invariant to monotonic grayscale changes and efficient at capturing micro-textures. GLCM, on the other hand, is a statistical method that characterizes how often pairs of pixel values occur in specific spatial relationships, enabling the extraction of second-order texture statistics such as contrast, homogeneity, correlation, and energy.

In digital forensics, these texture-based descriptors have shown promise in revealing hidden inconsistencies or artifacts introduced by image manipulation or synthesis (Xu and Shi, 2012). For instance, forged regions may exhibit subtle textural discontinuities or lack the natural statistical distribution of real images. By incorporating LBP and GLCM into forensic pipelines, researchers have been able to identify tampering traces that are not easily captured by semantic-level detectors. These methods provide an interpretable and complementary perspective to data-driven deep models, especially in low-data or high-risk scenarios.

### 2.3 Detecting AI-generated images

The detection of AI-generated images has attracted increasing attention in recent years (Lin et al., 2024a; Zhou et al., 2025), leading to a wide spectrum of proposed methods. Early techniques relied on handcrafted features such as noise residuals, color anomalies, or JPEG compression artifacts to identify inconsistencies introduced during image synthesis (Grommelt et al., 2025). However, these approaches often lacked robustness when confronted with diverse generative models or post-processing operations.

With the rise of deep learning, end-to-end convolutional neural networks (CNNs) have become the dominant approach in generative image detection. These models are trained to distinguish real from synthetic content directly from pixel-level data, leveraging their capacity to automatically learn discriminative features (Cozzolino et al., 2024). Recent work has further incorporated frequency-domain analysis (e.g., FFT, DCT) to capture spectral artifacts left by synthesis models, and transformer-based architectures have been explored for their long-range modeling abilities (Zhou et al., 2023).

Another active direction is multi-modal and hybrid detection, where different representations—such as semantic features (Ye et al., 2024), frequency cues, and residuals—are fused to improve robustness. Some studies have also explored contrastive learning, attention mechanisms, and domain adaptation to enhance generalization to unseen generators.

Multi-modal detection methods often integrate complementary features—such as spatial, frequency, and semantic information—to better capture subtle generative artifacts. For example, Li et al. (2022) proposed a dual-branch network that fuses spatial features with frequency-aware attention maps to improve the detection of GAN-generated images. Similarly, Zhao et al. (2021) incorporated semantic embeddings from CLIP along with visual textures to boost generalization across domains. These approaches leverage diverse feature streams to compensate for weaknesses in any single modality.

In parallel, frequency domain analysis has become a powerful tool in generative content detection. GANs often introduce abnormal frequency patterns due to upsampling and convolution artifacts, which are not always visible in the spatial domain. Methods such as Zhang et al. (2019) use Discrete Fourier Transform (DFT) representations to highlight high-frequency inconsistencies, while others apply Discrete Cosine Transform (DCT) or Wavelet transforms to extract compact yet discriminative features. More recently, phase-aware techniques have emerged that analyze the phase spectrum of images, which remains more stable under post-processing than magnitude components. For instance, Qian et al. (2023) demonstrate that phase-based residuals can expose subtle inconsistencies introduced by diffusion models and face reenactment systems. In recent years, multimodal large language models can be also adopted for detecting AI-generated images (He et al., 2025).

Despite notable progress, several limitations remain. First, most deep models rely heavily on large-scale labeled datasets, which may not cover all generative techniques and domains. Second, many detectors focus predominantly on semantic or content-level discrepancies, while neglecting subtle textural cues that may better reveal synthesis patterns. Third, the black-box nature of end-to-end learning hinders interpretability and increases vulnerability to adversarial attacks or domain shifts.

These limitations motivate the need for detection frameworks that can integrate interpretable and complementary information sources—such as texture semantics—alongside conventional visual features to improve accuracy, robustness, and generalization in real-world settings.

## 3 Proposed method

### 3.1 Overview of the framework

To effectively detect AI-generated images and uncover subtle synthesis artifacts, we propose a multi-branch convolutional neural network that leverages both semantic and texture-based information. The core idea is to extract and integrate multi-modal features from three complementary representations of the input image:

(1) The original RGB image, which preserves semantic content and color distribution;
(2) A Local Binary Pattern (LBP) representation, which captures local micro-textures and structural changes, note that we apply original LBP here that the texture features captured are in 256 dimensions;
(3) A Gray-Level Co-occurrence Matrix (GLCM) map, which encodes second-order statistical relationships between pixel intensities, we do not apply any pre-processing here but the grayscale conversion.

Each representation is fed into an individual CNN branch, enabling the network to learn modality-specific features. The three feature streams are subsequently fused and processed jointly to perform the final classification.

This design encourages the network to look beyond semantic cues and attend to hidden visual inconsistencies that are often embedded in textural patterns—an aspect commonly overlooked by standard end-to-end models.

### 3.2 Input representations and preprocessing

In our proposed framework, we construct a tri-modal input representation to enhance the networks ability to capture both semantic and fine-grained texture information. Specifically, each image is transformed into three distinct modalities: RGB, LBP, and GLCM, which are processed in parallel by three independent branches. The preprocessing procedures for each input channel are as follows:

RGB channel: the original RGB image is used to preserve high-level semantic content, including color distributions, object boundaries, and natural context. It serves as the baseline modality for learning visually discriminative features from unaltered pixel intensities.
LBP channel with edge-guided enhancement: to better highlight the structural inconsistencies often introduced in synthetic images, we introduce an edge-guided enhancement mechanism prior to computing Local Binary Patterns (LBP). Specifically, we first apply a classical edge detector, the canny operator, to locate prominent structural transitions in the image. The resulting edge map is then used to guide the selection of LBP regions—only pixels along or near the detected edges are retained for LBP encoding. This selective process focuses the LBP feature extraction on areas most likely to reveal unnatural transitions, suppressing noise in flat or homogeneous regions and improving the interpretability and relevance of the extracted micro-textures.
GLCM channel: for the third modality, we compute the Gray-Level Co-occurrence Matrix (GLCM) based on the grayscale version of the input image. Converting color images to grayscale ones are applied with the equation below.

(1) $$\begin{array}{l} Gray=0.299*R+0.587*G+0.114*B, \end{array}$$

where *R*, *G*, and *B* are pixels values in each channels.

GLCM captures second-order statistics by measuring the frequency of co-occurring pixel intensity pairs in a defined spatial relationship. From the GLCM, we derive texture descriptors such as contrast, correlation, and homogeneity. These descriptors are normalized and assembled into a feature map that reflects spatial texture dependencies, enriching the networks understanding of underlying statistical patterns.

By jointly leveraging these three representations, the model can integrate information from multiple perceptual levels—global semantics, local structure, and statistical texture—resulting in a more robust and explainable detection strategy.

### 3.3 Network architecture

**Figure 1:**
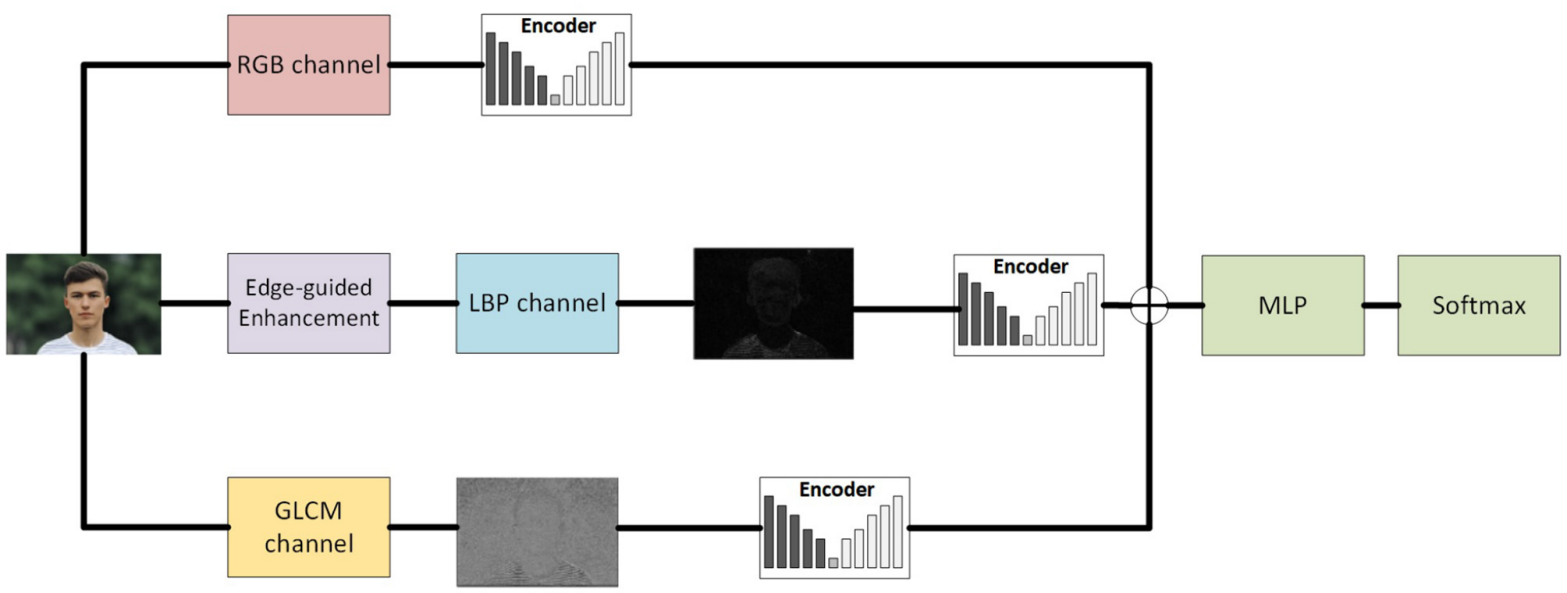
Architecture of the proposed model. Images are AI-generated.

The architecture of our proposed detection model is designed to extract and integrate multi-modal features through three parallel auto-encoder branches, followed by a unified fusion and classification module. Each branch is dedicated to one modality—RGB, edge-guided LBP, or GLCM—and is responsible for capturing unique semantic or texture-based cues from the input. The overall architecture is illustrated in Figure 1.

#### 3.3.1 Modality-Specific feature extraction via auto-encoders

Each input modality is processed through a dedicated auto-encoder consisting of an encoder-decoder pair. The encoder learns a compact, high-level representation of the input, while the decoder is used only during training for regularization purposes (e.g., reconstruction loss), encouraging the encoder to retain meaningful features.

The RGB branch encoder captures global visual semantics such as color consistency, object coherence, and scene layout.

The LBP branch, which receives an edge-enhanced LBP map as input, focuses on local micro-textures and structural discontinuities—particularly around image boundaries where synthetic inconsistencies often emerge.

The GLCM branch encoder extracts statistical texture representations based on co-occurrence patterns that are indicative of synthetic regularities or unnatural smoothness.

Each encoder consists of a series of convolutional layers, normalization, and non-linear activation functions. The output from the final convolutional block in each encoder is flattened into a feature vector representing the modality-specific embedding.

#### 3.3.2 Feature fusion and classification

The three modality-specific feature vectors are concatenated to form a unified representation. It is a straightforward concatenation, directly combine the feature of all channels together. This fused feature vector is then fed into a multi-layer perceptron (MLP) classifier composed of fully connected layers with ReLU activations and dropout regularization. Finally, a softmax layer produces the probability distribution over the binary class labels (real vs. AI-generated).

This architecture allows each branch to learn and preserve distinct types of forensic cues, while the joint classifier integrates these complementary features to make an informed prediction. The auto-encoder-based design also facilitates future extension to unsupervised or self-supervised training paradigms.

### 3.4 Feature fusion and classification

Once modality-specific features have been extracted by the three auto-encoder branches, the next step is to effectively integrate this multi-modal information and make a final classification. The fusion and classification module is designed to preserve the complementary strengths of each channel while enhancing the overall discriminative capacity of the model.

The output feature vectors from the RGB, edge-guided LBP, and GLCM encoders are first flattened and then concatenated along the feature dimension to form a single joint representation. Formally, let *f*_RGB_, *f*_LBP_, and *f*_GLCM_ denote the features extracted from the respective branches. The fused representation is obtained as:


(2) $$\begin{array}{l} f_{\text{fusion}}=\left[f_{\text{RGB}}||f_{\text{LBP}}||f_{\text{GLCM}}\right] \end{array}$$

This straightforward concatenation strategy ensures that the network retains the full scope of information learned from each modality. In practice, this joint vector contains both global semantic cues and fine-grained textural features that may independently or jointly reveal inconsistencies caused by AI synthesis.

The fused feature vector *f*_fusion_ is passed through a multi-layer perceptron (MLP) composed of two or more fully connected (FC) layers. Each FC layer is followed by a non-linear activation function (e.g., ReLU) and dropout layers to prevent overfitting. The final FC layer outputs a two-dimensional vector, which is then passed through a softmax function to produce class probabilities:


(3) $$\begin{array}{l} p=\text{softmax}\left(W\cdot f_{\text{fusion}}+b\right) \end{array}$$

where *W* and *b* are the weights and biases of the final classification layer, and *p*∈ℝ^2^ denotes the probability of the input being either real or AI-generated.

An important advantage of this design is its interpretability and modularity. Since each input modality has a dedicated branch, it is possible to visualize and analyze the individual contributions of semantic and texture-based features. Furthermore, this modular structure allows future integration of additional modalities or alternate fusion strategies, such as attention-based weighting or gating mechanisms.

In summary, our fusion and classification design maximizes the synergy between diverse input features, resulting in a more robust and generalizable detection framework.

### 3.5 Training strategy

To effectively train the proposed multi-branch detection network, we adopt a supervised learning framework based on cross-entropy loss. The training process is designed to encourage each modality-specific encoder to capture discriminative features, while the classifier learns to make robust predictions based on fused multi-modal information.

The primary objective is to correctly classify whether an input image is real or AI-generated. We use the standard cross-entropy loss:


(4) $$\begin{array}{l} L_{\text{cls}}=-\overset{C}{\underset{i=1}{\sum }}y_{i}\log\left(p_{i}\right) \end{array}$$

where *C* = 2 denotes the number of classes (real vs. fake), *y*_*i*_ is the ground truth label (one-hot encoded), and *p*_*i*_ is the predicted probability output from the softmax layer.

If reconstruction supervision is used for the auto-encoders, an auxiliary reconstruction loss $L_{\text{rec}}$ can be added to the overall objective to encourage modality-preserving feature extraction:


(5) $$\begin{array}{l} L_{\text{rec}}=\underset{m\in \left\{\text{RGB},\text{LBP},\text{GLCM}\right\}}{\sum }||I_{m}-{Î}_{m}|{|}_{2}^{2} \end{array}$$

where *I*_*m*_ and Î_*m*_ denote the original and reconstructed images in modality *m*, respectively.

The total loss becomes:


(6) $$\begin{array}{l} L=L_{\text{cls}}+\lambda L_{\text{rec}} \end{array}$$

where λ is a hyperparameter controlling the contribution of the reconstruction loss (set to 0 if no reconstruction loss is used).

The model is trained using the Adam optimizer with the following hyperparameters: learning rate 1 × 10^−4^, batch size 32, and weight decay 5 × 10^−5^. Training is conducted for 50 epochs with early stopping based on validation accuracy.

To enhance generalization, standard data augmentation techniques such as random cropping, horizontal flipping, and color jittering are applied to the RGB channel. For the LBP and GLCM branches, input normalization is used instead of geometric transformations to preserve texture fidelity.

## 4 Experiments

In this section, we conduct extensive experiments to evaluate the effectiveness of our proposed multi-modal detection framework. The experiments are designed to assess not only the overall classification performance but also the contribution of individual modalities and the effect of the proposed enhancements.

### 4.1 Experimental setup

#### 4.1.1 Datasets

To evaluate the effectiveness and generalizability of our proposed detection method, we conduct experiments on two representative benchmark datasets: ForenSynths and GenImage. The ForenSynths dataset is a large-scale benchmark specifically curated for forensic analysis of AI-generated content. It consists of both real and synthetically generated images across diverse semantic categories, collected using multiple generative models such as StyleGAN, BigGAN, and DALL·E. Each image is paired with corresponding metadata and pixel-level annotations to facilitate localization and classification tasks. ForenSynths is widely used in deepfake detection and digital forensics research due to its diversity and fine-grained annotations.

The GenImage dataset is a more recent benchmark designed to assess the robustness of detectors against a wide spectrum of generative models and post-processing conditions. It contains a large number of images generated by cutting-edge diffusion models, transformer-based generators, and text-to-image systems like Stable Diffusion and Midjourney. GenImage emphasizes cross-model generalization, as it includes content from over 30 generative pipelines and simulates various real-world distortions, such as JPEG compression, resizing, and Gaussian noise. This makes it an ideal testbed for evaluating a detectors resilience to distribution shifts and unseen generators.

The FaceForensics++ (FF++) dataset is one of the most widely used benchmarks for deepfake detection, containing over 1,000 high-quality videos. It includes forged samples generated by multiple facial manipulation techniques, such as Face2Face, FaceSwap, DeepFakes, and NeuralTextures. FF++ also provides both raw videos and compressed versions at different levels, simulating distortions commonly encountered in real-world scenarios, which makes it highly valuable for training and benchmarking detection algorithms.

Celeb-DF is a more challenging dataset designed to address the limitations of earlier benchmarks where synthetic artifacts were too obvious. It contains over 5,900 high-resolution manipulated videos, most of which target publicly known celebrities. Compared with FF++, the forged videos in Celeb-DF are visually more realistic with fewer artifacts, making it closer to real-world application conditions.

WildDeepfake is collected directly from the internet, representing “in-the-wild” cases of AI-generated content. It consists of videos of diverse quality and sources, where forgeries are often less regular and harder to detect. Unlike FF++ and Celeb-DF, which are generated in controlled environments, WildDeepfake better reflects social media and online video platforms, providing a critical benchmark for evaluating the generalization ability of detection models.

Together, these datasets provide a comprehensive and challenging environment for benchmarking the robustness, generalization, and fine-grained discriminability of AI-generated image detection methods.

#### 4.1.2 Evaluation metrics

We use standard binary classification metrics to evaluate model performance:

- Accuracy (Acc): overall proportion of correctly classified samples.
- Precision (Prec): ratio of true positives among predicted positives.

#### 4.1.3 Implementation details

The model is implemented in PyTorch and trained on a single NVIDIA RTX 3090 GPU. All input images are resized to 256 × 256. For each image, three input branches are constructed: RGB, LBP-enhanced edge map, and GLCM-based texture representation. Hyperparameters include:

- Optimizer: adam
- Learning rate: 1 × 10^−4^
- Batch size: 32
- Epochs: 50 (with early stopping)

Data augmentations applied to the RGB input include random horizontal flipping, cropping, and color jittering. For LBP and GLCM channels, input normalization is used to retain texture consistency.

### 4.2 Overall performance

We evaluate the full version of our proposed multi-modal detection framework on ForenSynths (Wang et al., 2020). The models are trained with the ProGAN (Karras, 2017) dataset, then evaluated with other methods, including various GANs. Also the models are tested with GenImage (Dhariwal and Nichol, 2021) to evaluate the capability for discerning images generated by diffusion models.

Several state-of-the-art methods for detecting AI-generated images are chosen as baselines for comparison. Wang et al. (2020) proposes to train a CNN model for general AI-generated image detection. Liu et al. (2022) designs a noise model to expose AI-generated images from the frequency components. Ojha et al. (2023) adopts a pre-trained CLIP for distinguishing images generated by AI. Zhou et al. (2023) is a method to expose deepfakes via ViT. Tan et al. (2024) employs the neighbor pixel relationships for capturing the traces left by upsampling in AI-generated images. Deressa et al. (2025) detecting deepfakes using a generative convolutional vision transformer. Tables 1–3 summarize the classification results in terms of detection accuracy, average precision, and AUC.

**Table 1:** Overall detection performance of accuracy and average precision on ForenSynths dataset.

**Method**	**ProGAN**	**StyleGAN**	**BigGAN**	**StarGAN**	**CycleGAN**
Wang et al. (2020)	100.0/100.0	87.1/99.6	70.2/84.5	91.7/98.2	85.2/93.5
Liu et al. (2022)	99.9/100.0	92.6/99.2	88.1/95.2	100.0/100.0	79.0/89.5
Ojha et al. (2023)	100.0/100.0	91.0/98.3	94.5/91.3	97.0/99.8	98.5/99.4
Zhou et al. (2023)	100.0/100.0	95.7/98.7	94.1/93.4	98.6/99.2	95.5/96.3
Tan et al. (2024)	99.8/100.0	96.3/99.8	87.5/94.5	99.7/100.0	95.0/99.5
Deressa et al. (2025)	99.9/100.0	96.3/98.8	94.6/95.2	99.5/99.7	97.7/98.4
Ours	100.0/100.0	96.5/99.8	95.2/98.6	100.0/100.0	98.5/99.5

All models are trained with ProGAN and tested with others.

**Table 2:** Overall detection performance of AUC on ForenSynths dataset.

**Method**	**ProGAN**	**StyleGAN**	**BigGAN**	**StarGAN**	**CycleGAN**
Wang et al. (2020)	99.9	88.3	71.3	92.3	87.5
Liu et al. (2022)	99.9	91.7	90.0	99.9	82.3
Ojha et al. (2023)	99.9	93.7	94.0	97.3	98.7
Zhou et al. (2023)	99.9	94.0	94.2	97.0	98.5
Tan et al. (2024)	99.8	96.9	89.9	99.8	96.45
Deressa et al. (2025)	99.9	96.5	95.9	98.7	97.6
Ours	99.9	97.2	96.1	99.9	98.8

All models are trained with ProGAN and tested with others.

**Table 3:** Overall detection performance on GenImage dataset.

**Method**	**Midjourney**	**SDv1.4**	**SDv1.5**	**Wukong**
Wang et al. (2020)	50.8/58.6/57.2	51.1/59.2/58.5	51.2/59.9 /60.1	51.0/57.0 /55.4
Liu et al. (2022)	52.0/58.2/57.7	54.2/60.1/ 59.5	65.3/68.6/66.7	58.0/68.1 /62.3
Ojha et al. (2023)	56.1/74.0 /60.2	63.7/86.1/67.7	63.5/85.8 /67.5	85.3/96.5/88.2
Zhou et al. (2023)	70.3/79.5/ 77.8	76.8/83.3/ 79.5	80.2/81.9/ 81.3	80.3/88.6/85.7
Tan et al. (2024)	78.0/85.6/79.9	78.9/84.2/81.3	79.0/84.9/79.2	76.3/80.7/77.4
Deressa et al. (2025)	87.2/87.6/ 86.0	82.1/87.1/84.1	74.6/80.3/ 80.1	83.9/82.5/ 83.3
Ours	88.2/90.1/88.9	86.5/89.3/89.0	92.1/95.4/ 93.9	86.3/88.8 /88.7

All models are trained with ProGAN and tested with others.

Other than these results, we also evaluate the proposed model with deepfake datasets. Adhered from the classicial manner, all models are trained with FF++ and tested with samples from FF++, Celeb-DF, wilddeepfake. The generalizability can be also examined via this design. The results are reported in Table 4.

**Table 4:** Overall detection performance on deepfake dataset.

**Method**	**FF++**	**Celeb**	**wilddeepfake**
Wang et al. (2020)	90.5/91.2	63.2/60.7	59.7/59.0
Liu et al. (2022)	93.5/94.4	64.5/63.7	64.3/66.2
Ojha et al. (2023)	94.1/94.0	72.4/73.1	73.9/75.6
Zhou et al. (2023)	97.7/96.5	80.5/82.3	78.4/78.6
Tan et al. (2024)	97.8/97.2	78.1/76.3	79.2/77.5
Deressa et al. (2025)	98.5/97.0	80.9/82.4	83.6/84.7
Ours	99.3/99.1	83.5/84.1	85.6/84.8

All models are trained with FF++ and tested with others. The results are reported with accuracy and AUC.

The results demonstrate that our model achieves strong performance across all metrics and datasets. The model effectively distinguishes between real and AI-generated images even under varying synthesis techniques.

Also, observed from the tables, there is a distinct performance difference. When a detection model is trained primarily on GAN-generated images, it may not generalize well to images generated by diffusion models. This is because different generative architectures introduce distinct types of artifacts and visual patterns. GANs often produce local texture inconsistencies or checkerboard artifacts due to upsampling, while diffusion models tend to generate globally coherent but subtly unnatural image structures. As a result, a model that learns to detect the typical traces of GANs might struggle to identify the less obvious or differently distributed artifacts in diffusion-based images, leading to a performance gap across generation types.

**Figure 2:**
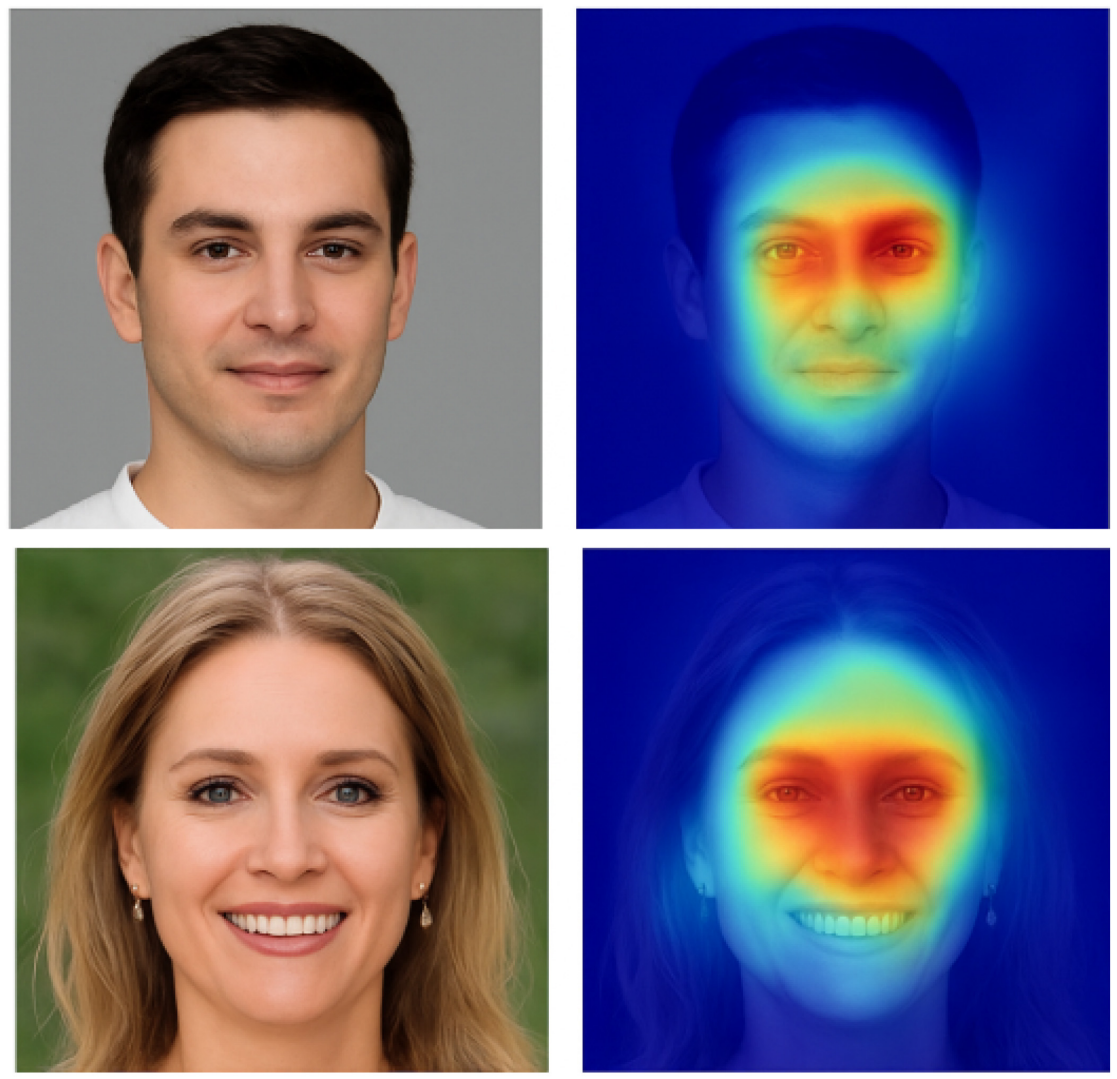
Grad-CAM visualization of samples. Images are AI-generated.

Compared to existing arts, the integration of texture-based features through LBP and GLCM enhances the model's sensitivity to subtle inconsistencies in AI-generated content. The performance on FF++, which contains multiple manipulation types, suggests the generalization capacity of our approach. Visualizations of two samples are displayed in Figure 2.

### 4.3 Ablation study

To better understand the contribution of each modality and component in our proposed network, we conduct a set of ablation experiments. We evaluate the following variants:

- RGB only: using only the original RGB image channel.
- RGB + LBP: combining RGB with the LBP-enhanced edge representation.
- RGB + GLCM: combining RGB with the GLCM-based texture representation.
- Full (RGB + LBP + GLCM): our complete proposed model using all three modalities.
- Full w/o edge enhancement: removing the edge extraction step before LBP in the full model.

Table 5 summarizes the performance of each variant. All models are trained with ProGAN and tested with BigGAN.

**Table 5:** Ablation study results.

**Variant**	**Accuracy**	**Average Precision**	**AUC**
RGB only	88.7%	89.2%	87.1%
RGB + LBP	91.2%	91.8%	89.9%
RGB + GLCM	91.9%	92.5%	90.2%
Full (RGB + LBP + GLCM)	**95.2%**	**98.6%**	**97.8%**
Full w/o Edge Enhancement	92.0%	92.9%	92.1%

The bold values indicate the values of best performance for comparisons.

From the results, we observe the following:

- Adding LBP or GLCM branches to RGB improves performance, validating the utility of texture features.
- The complete model (RGB + LBP + GLCM) outperforms all variants, confirming that the multi-modal fusion contributes complementary discriminative features.
- Removing the edge enhancement step in LBP results in a noticeable drop in performance, which highlights the importance of performing LBP on semantically rich edge regions rather than the entire image.

These findings demonstrate that carefully crafted texture feature extraction, especially the proposed edge-guided LBP representation, plays a crucial role in enhancing the networks ability to detect AI-generated image artifacts.

### 4.4 Robustness evaluation

To further evaluate the practical applicability of our proposed method, we conduct robustness experiments by introducing common image perturbations that often occur during post-processing or real-world transmission. Specifically, we assess the model's performance under the following distortions:

- JPEG compression: quality factor reduced to 50.
- Gaussian blur: applied with a kernel size of 5 × 5 and standard deviation of 1.5.
- Image sharpening: using a Laplacian-based kernel.
- Adversarial attack: applying the adversarial attack proposed in Ding et al. (2021).

Table 6 presents the detection performance (Accuracy and AUC) of the full model on the Celeb-DF v2 dataset after applying each distortion.

**Table 6:** Robustness evaluation under common image distortions on BigGAN.

**Perturbation**	**Accuracy**	**AP**	**AUC**
Original (clean)	95.2%	98.6%	96.4%
JPEG compression (*Q*= 50)	89.1%	94.0%	92.1%
Gaussian blur	87.6%	92.8%	91.5%
Image sharpening	91.0%	95.3%	92.3%
Adversarial attack	83.4%	82.9%	80.8%

The results demonstrate that while there is a modest degradation in detection performance under perturbations, our model maintains relatively high accuracy and AP values, particularly under compression and sharpening. This robustness can be attributed to the incorporation of texture-based features, which are less sensitive to global color shifts or pixel-level noise.

Among the three perturbations, Gaussian blur causes the largest performance drop, likely because it removes high-frequency artifacts that are essential for forgery detection. Nevertheless, even under this scenario, the model still achieves an accuracy of 87.6%, which underscores the resilience of our feature extraction scheme.

These findings indicate that our method can generalize well to real-world conditions where image degradation is inevitable, making it suitable for practical forensic applications.

### 4.5 Interpretation

We observe the following:

- For real images, the model distributes its attention more evenly across the facial region, suggesting natural texture consistency.
- For AI-generated images, the model tends to focus on high-frequency regions such as eyes, mouth contours, and facial edges, which often contain subtle synthesis artifacts.
- These findings support our hypothesis that texture-based features—especially those emphasized by LBP and GLCM—help highlight micro-level irregularities that are not apparent in raw RGB inputs.

These qualitative results verify that each modality captures complementary aspects of the forgery, and that the model learns to localize regions with anomalous texture patterns, which are often indicative of AI-generated artifacts.

## 5 Conclusion

In this paper, we proposed a novel multi-modal framework for the detection of AI-generated images by incorporating texture-aware representations into a three-branch network.

Specifically, we introduced an edge-guided LBP branch that extracts local binary features along semantically salient boundaries, and a GLCM branch that models statistical texture correlations. Each input modality is processed via an independent auto-encoder network, and the fused feature representations are passed through a multilayer perceptron for final classification. Our design encourages the model to capture complementary information across multiple input types, improving generalization to unseen forgeries.

Extensive experiments on standard benchmarks demonstrate the effectiveness of our approach. The proposed method outperforms several baseline and state-of-the-art detectors. Ablation studies confirm the individual contributions of the LBP and GLCM modalities, as well as the importance of edge-aware preprocessing.

In the future, we plan to extend our work by incorporating frequency-domain and temporal information to handle more complex video-based forgeries. We also aim to explore lightweight variants of our model for deployment in real-time applications. While these extensions may require additional computational resources—especially when integrating high-dimensional frequency cues or long-range temporal dependencies—the recent advances in GPU acceleration, edge AI devices, and model compression techniques (e.g., pruning, quantization, and knowledge distillation) provide a promising pathway for practical deployment. Thus, we believe that with careful algorithm-hardware co-design, our proposed framework and its future variants are feasible to be deployed in real-world scenarios such as financial fraud detection, digital media verification, and online content monitoring.

## Data Availability

The raw data supporting the conclusions of this article will be made available by the authors, without undue reservation.
